# A surge of late-occurring meiotic double-strand breaks rescues synapsis abnormalities in spermatocytes of mice with hypomorphic expression of SPO11

**DOI:** 10.1007/s00412-015-0544-7

**Published:** 2015-10-06

**Authors:** Monica Faieta, Stefano Di Cecca, Dirk G. de Rooij, Andrea Luchetti, Michela Murdocca, Monica Di Giacomo, Sara Di Siena, Manuela Pellegrini, Pellegrino Rossi, Marco Barchi

**Affiliations:** Department of Biomedicine and Prevention, Section of Anatomy, University of Rome Tor Vergata, 00133 Rome, Italy; Reproductive Biology Group, Division of Developmental Biology, Department of Biology, Faculty of Science, Utrecht University, Utrecht, Netherlands; Center for Reproductive Medicine, Academic Medical Center, University of Amsterdam, Meibergdreef 9, 1105 AZ Amsterdam, The Netherlands; Department of Biomedicine and Prevention, Section of Genetics, University of Rome Tor Vergata, 00133 Rome, Italy; European Molecular Biology Laboratory (EMBL), Monterotondo, Italy; DAHFMO, Sapienza University, Rome, Italy; Department of Medicine and Health Science “Vincenzo Tiberio”, University of Molise, Campobasso, Italy

**Keywords:** SPO11, Chromosome synapsis, Double-strand breaks (DSBs), Meiotic recombination, Meiosis, Spermatogenesis

## Abstract

Meiosis is the biological process that, after a cycle of DNA replication, halves the cellular chromosome complement, leading to the formation of haploid gametes. Haploidization is achieved via two successive rounds of chromosome segregation, meiosis I and II. In mammals, during prophase of meiosis I, homologous chromosomes align and synapse through a recombination-mediated mechanism initiated by the introduction of DNA double-strand breaks (DSBs) by the SPO11 protein. In male mice, if SPO11 expression and DSB number are reduced below heterozygosity levels, chromosome synapsis is delayed, chromosome tangles form at pachynema, and defective cells are eliminated by apoptosis at epithelial stage IV at a spermatogenesis-specific endpoint. Whether DSB levels produced in *Spo11*^+/−^ spermatocytes represent, or approximate, the threshold level required to guarantee successful homologous chromosome pairing is unknown. Using a mouse model that expresses *Spo11* from a bacterial artificial chromosome, within a *Spo11*^−/−^ background, we demonstrate that when SPO11 expression is reduced and DSBs at zygonema are decreased (approximately 40 % below wild-type level), meiotic chromosome pairing is normal. Conversely, DMC1 foci number is increased at pachynema, suggesting that under these experimental conditions, DSBs are likely made with delayed kinetics at zygonema. In addition, we provide evidences that when zygotene-like cells receive enough DSBs before chromosome tangles develop, chromosome synapsis can be completed in most cells, preventing their apoptotic elimination.

**Electronic supplementary material:**

The online version of this article (doi:10.1007/s00412-015-0544-7) contains supplementary material, which is available to authorized users.

## Introduction

Meiosis is the specialized cellular process through which haploid gametes are formed for sexual reproduction. In mammals, spermatogenesis takes place within the seminiferous tubules, in which mitotic spermatogonia divide, differentiate, and subsequently enter meiotic divisions. During prophase of meiosis I, homologous chromosomes from differing parental origin (each consisting of two sister chromatids), pair, and synapse. Following the formation of physical links (chiasmata), they subsequently align and move to opposite poles at metaphase I. At the second meiotic division, sister chromatids separate to form haploid round spermatids, which elongate and eventually mature into spermatozoa (Hamer et al. 2008). Failure of homologous synapsis elicits the activation of a male-specific meiotic checkpoint, which eliminates defective cells via apoptosis, thereby preventing the formation of gametes with damaged or aneuploid genomes (de Rooij and de Boer 2003; Barchi et al. 2005; Hamer et al. 2008; Pacheco et al. 2015).

In mammals, as in many sexually reproducing organisms, proper meiotic chromosome synapsis and the formation of chiasmata depend on the initiation of the recombination process that is induced by the generation of SPO11-mediated double-strand breaks (DSBs) (Keeney 2001, 2008). Subsequent to their formation, DSB ends are repaired by homologous recombination, ultimately giving rise to chromosomal pairing and formation of crossovers (CO), that is, interhomolog DNA links, cytologically identifiable as chiasmata (Keeney 2001, 2008).

During prophase I, the recombination-mediated establishment of stable interaction between homologous chromosomes (henceforth referred to as homologs) is a progressive process. It initiates at leptonema with chromosomal pairing (that is, close juxtaposition between homologs) and proceeds during zygonema, leading to alignment of homologs, whose physical interaction is stabilized by the formation of a zipper-like proteinaceous structure called the synaptonemal complex (SC) (Page and Hawley 2004). In mammals, as in other organisms that rely on recombination for efficient pairing, DSB-mediated chromosomal interactions initiate at multiple sites along their length. For this reason, SPO11-mediated DSB numbers largely exceed that of crossovers, and DSBs are made at multiple locations [see (Keeney et al. 2014) and references therein].

In both mice and humans, the *Spo11* gene encodes two major splice variants, which differ by the inclusion (SPO11β) or exclusion (SPO11α) of exon 2 (Keeney et al. 1999; Romanienko and Camerini-Otero 1999; Bellani et al. 2010). Another splicing event occurs between exons 7 and 8, with the generation of alternative isoforms of Spo11β and Spo11α known as *bclI* and *sphI*, respectively (Keeney et al. 1999; Romanienko and Camerini-Otero 1999; Bellani et al. 2010). These isoforms are differentially expressed by spermatocytes, during prophase I: SPO11β mRNA peaks in early prophase, when DSBs are made at leptonema and zygonema. SPO11α appears at zygonema and accumulates in late prophase (pachynema to diplonema stages), when most of the DSBs generated at leptonema have been already repaired and chromosomal pairing has been achieved (Bellani et al. 2010). Complementation of *Spo11*^−/−^ by the ectopic expression of SPO11β^bclI^ under the control of *Xmr* promoter revealed that, when this splicing isoform is expressed at wild-type levels, it supports proper DSB formation and synapsis of all chromosomes, with the exception of the XY pair (Kauppi et al. 2011, 2013a). In contrast, when SPO11β^bclI^ expression is reduced relative to that of *Spo11*^+/−^ mice, proper synapsis of short autosomes and sex chromosomes fails, with the successive formation of chromosome tangles at pachynema (Kauppi et al. 2013a). This finding suggested the existence of an as-of-yet unidentified threshold level of DSBs required to guarantee successful chromosome synapsis (Kauppi et al. 2013a, b). In wild-type mice, an estimated average ~140 DSBs are made at the onset of recombination process during leptonema (Cole et al. 2012). This number increases (DSB surge) when cells progress to early/mid-zygonema (~200 foci per cell), likely in an attempt to enforce the homologous search process (Cole et al. 2012). When *Spo11* gene dosage is halved, DSBs are ~20 % reduced, at zygonema (Bellani et al. 2010; Cole et al. 2012). Beside this, *Spo11*^+/−^ mice are phenotypically normal (Bellani et al. 2005; Cole et al. 2012; Kauppi et al. 2013a), indicating that in this mouse, SPO11-generated DSB number at zygonema is above the threshold level for proper progression of chromosome paring/synapsis. Whether DSBs produced in *Spo11*^+/−^ mice represent the threshold level for mammalian meiosis is unknown, due to the lack of additional genetic models to further titrate DSB numbers.

In order to develop a genetic tool to delete early meiotic genes, we generated a mouse line in which both SPO11 and the CRE-recombinase were expressed by a bacterial artificial chromosome (BAC), under the *Spo11* promoter (*Spo11*-IRES-*Cre* mice) [see Supplemental Fig. 1 and (Pellegrini et al. 2011)]. The analysis of CRE-recombinase activity in vivo revealed that *Cre* expression (driven by *Spo11* promoter) was able to delete a post-meiotic gene, recapitulating the phenotype of the knockout mouse model. However, when Spo*11*-IRES-*Cre* mice were used to delete a gene with an early meiotic function [namely *Nbs1* (Difilippantonio et al. 2005)], the deletion only partially recapitulated the expected phenotype. From this observation, we reasoned that either the *Cre*-recombinase was correctly expressed but did not reach the critical level needed for *Nbs1* deletion by early stages of meiosis, or *Cre* expression (and that of SPO11) was delayed/reduced in early meiosis. According to the latter hypothesis, this mouse model could potentially provide a tool to study effects of changes in SPO11 expression timing or levels, on meiotic chromosome dynamics in vivo.

Using mice expressing the *Spo11*-IRES-*Cre* construct on a *Spo11*^−/−^ background, we have now found that when spermatocytes receive an approximately 40 % reduction in DSB dosage at zygonema, chromosome synapsis progresses normally. In addition, we provided evidence that if cells with critically reduced numbers of DSBs receive a late surge of DSBs, their synaptic abnormalities are resolved, and thus spermatocytes progress through prophase I without relevant activation of the prophase I checkpoint.

## Results

### Expression of *Spo11*-IRES-*Cre* transgene almost fully complements *Spo11*^−/−^ mutation

**Fig. 1:**
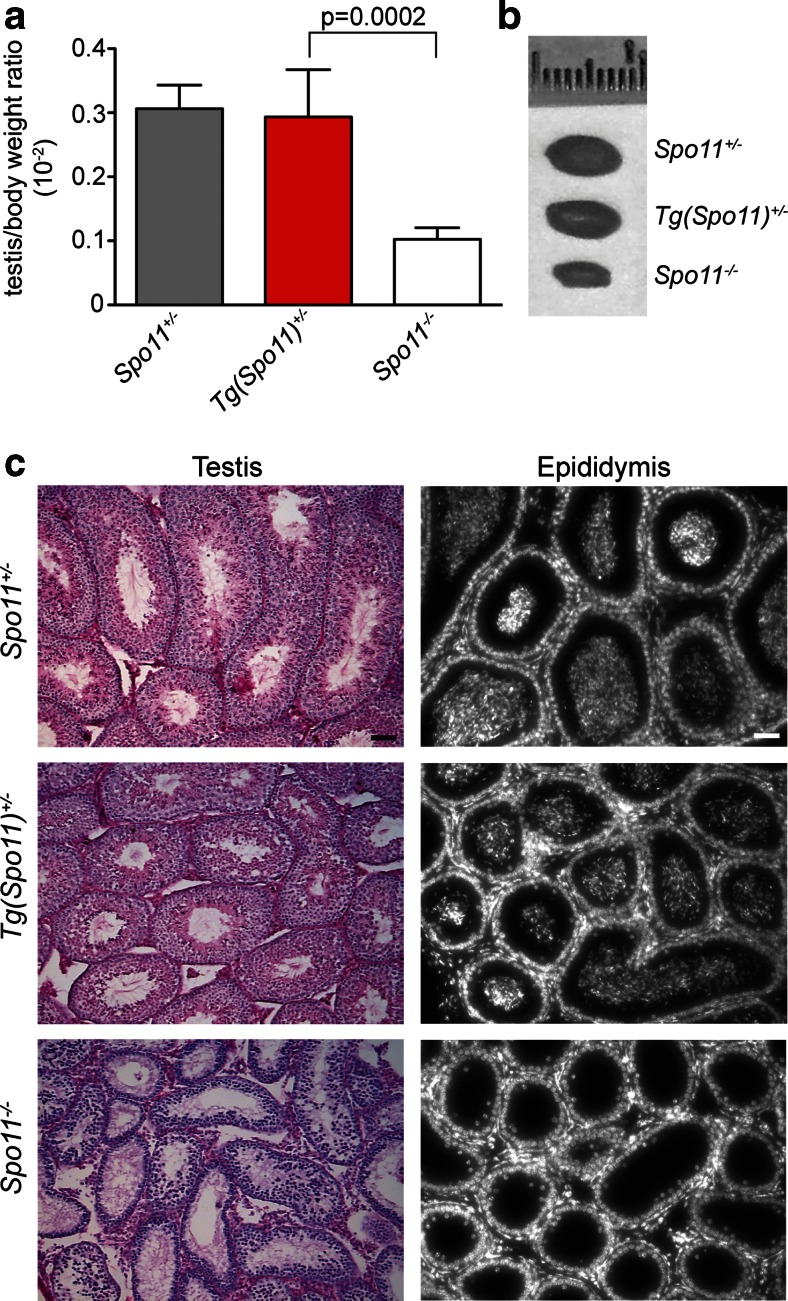
Histological analysis of testes and epididymis from *Spo11* ^+/−^, *Tg*(*Spo11*)^+/−^, and *Spo11* ^−/−^ mice. **a** Testis/body weight ratio in adult mice of the indicated genotypes (*Spo11* ^+/−^, *n* = 6 mice; *Tg*(*Spo11*)^+/−^, *n* = 9 mice; *Spo11* ^−/−^, *n* = 7 mice); *t* test *p* ≤ 0.05. **b** Representative image of the testis in the indicated genotypes. **c** Periodic acid-Schiff-stained testis cross sections from the indicated genotypes. Hoechst staining of epididymes from mice with the indicated genotypes. Spermatozoa were evident in the epididymis of *Spo11* ^+/−^ and *Tg*(*Spo11*)^+/−^ mice, while they were absent in *Spo11* ^−/−^ (*Spo11* ^+/−^, *n* = 3 mice; *Tg*(*Spo11*)^+/−^, *n* = 5 mice; *Spo11* ^−/−^, *n* = 3 mice). *Magnification bar* represents 50 μm. *Error bars* = mean ± SD

To understand whether expression of the *Spo11*-IRES-*Cre* transgene is able to complement *Spo11* mutation, we generated mice which expressed SPO11 exclusively from a hemizygous *Spo11*-IRES-*Cre* transgenic locus, henceforth referred to as *Tg*(*Spo11*)^+/−^. Testes from adult mice were analyzed histologically and compared to those of *Spo11*^−/−^ and *Spo11*^+/−^ littermates. The latter were used as controls, since this genotype was obtained with higher frequency than wild type and was reported to be phenotypically indistinguishable from *Spo11*^+/+^ (Bellani et al. 2005; Cole et al. 2012; Kauppi et al. 2013a). Weight and size of testis isolated from *Tg*(*Spo11*)^+/−^ mice were indistinguishable from that of *Spo11*^+/−^ littermates (Fig. 1a, b). In addition, histological analysis of testis sections revealed that, contrary to *Spo11*^−/−^ (Baudat et al. 2000; Romanienko and Camerini-Otero 2000), in *Tg*(*Spo11*)^+/−^ mice, seminiferous tubule composition and cellularity were similar to those in *Spo11*^+/−^ control testes (Fig. 1c). Germ cells progressed through the formation of morphologically mature sperms that did reach the epididymis (Fig. 1c) and the mice were fertile (supplemental Table 1). These observations suggested that the expression of SPO11 driven by the BAC-derived transgene fully complemented the *Spo11* mutation. However, a more detailed analysis of testis cross sections from the above genotypes revealed that a subset of seminiferous tubules of *Tg*(*Spo11*)^+/−^ mice presented abnormalities.

**Fig. 2:**
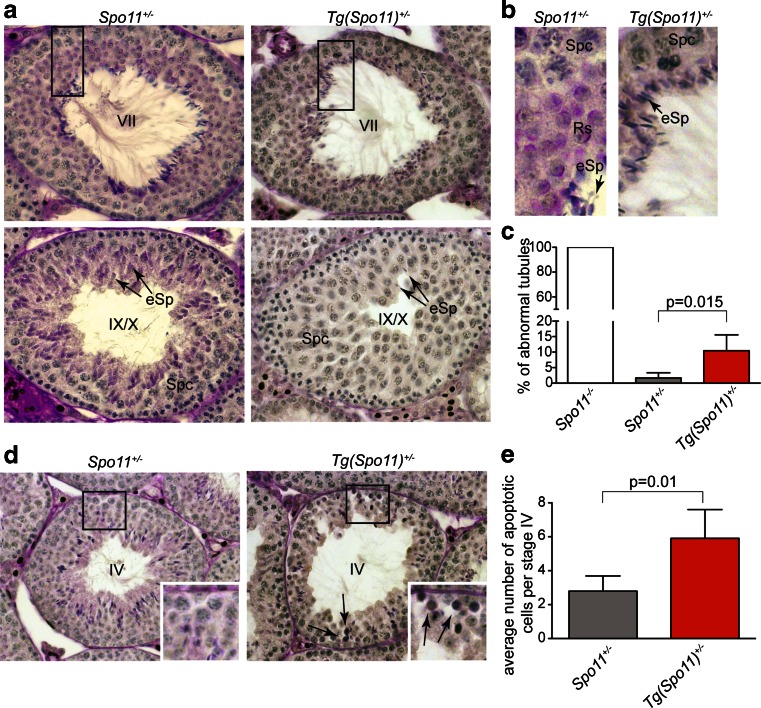
Analysis of histological abnormalities and apoptosis in testis cross sections of *Tg*(*Spo11*)^+/−^ and control testis. **a** Periodic acid-Schiff-stained testis sections from the indicated genotypes. *Top row*: comparison of testis cross sections in stage VII of the epithelial cell cycle. This seminiferous tubule of a *Tg*(*Spo11*)^+/−^ mouse lacks detectable round spermatids in more than half of the seminiferous tubule cross section, with consequent decrease of cellular density. **b** Enlargements showing that round spermatids (*Rs*) are always present in the population in *Spo11* ^+/−^, while Rs are missing in about half of this tubule of a *Tg*(*Spo11*)^+/−^ mouse. *Spc* = spermatocytes, *eSp* = elongated spermatids. *Bottom row*: comparison of testis cross sections in stage IX/X of the epithelial cycle. The tubule from a *Tg*(*Spo11*)^+/−^ mouse misses eSp. *Arrows* indicate eSp. **c** Quantification of total amount of tubules at any epithelial stage exhibiting abnormal histology [*Spo11* ^−/−^, *n* = 100 cross sections, one mouse analyzed; *Spo11* ^+/−^, *n* = 499 cross sections, three mice analyzed; *Tg*(*Spo11*)^+/−^, *n* = 900 cross sections, five mice analyzed]. *Error bars* = mean ± SD. *T* test on arcsin-transformed percentages, *p* ≤ 0.05. **d** Periodic acid-Schiff-stained testis sections of tubules at epithelial stage IV. *Arrows* indicate apoptotic cells. **d** Quantification of the average number of apoptotic cells per stage IV tubule [S*po11* ^+/−^, *n* = 110 stage IV tubule cross sections, *n* = 3 mice; *Tg*(*Spo11*) ^+/−^, *n* = 170 stage tubules cross sections, *n* = 5 mice]. Apoptotic cell number was significantly increased (*t* test, *p* ≤ 0.05) relative to the control. *Error bars* = mean ± SD

In mammals, each seminiferous tubule cross section can be assigned to one of 12 epithelial stages (numbered I–XII) based on the array of germ cell developmental stages that it contains (Russell et al. 1990; Ahmed and de Rooij 2009; Muciaccia et al. 2013). Within each epithelial stage, germ cell composition is established by the timely progression of germ cells during differentiation. Thus, the study of germ cell content and composition in each stage is informative in monitoring for defects in germ cell development and loss, over time. The analysis of testis sections from *Tg*(*Spo11*)^+/−^ mice uncovered that, as shown in Fig. 2a, b and quantified in Fig. 2c, a small but significant percentage (10.5 ± 5 %) of seminiferous tubules cross sections missed one or more germ cell generations in at least half of the seminiferous tubule, indicating that germ cell loss had occurred. In male mice, spermatocytes with defects in the timely resolution of DSBs and/or autosomal synapsis are eliminated by apoptosis at epithelial stage IV, which in wild-type spermatocytes corresponds to mid-pachytene stage (de Rooij and de Boer 2003; Barchi et al. 2005; Hamer et al. 2008; Mahadevaiah et al. 2008; Roig et al. 2010; Pacheco et al. 2015). On the contrary, if cells complete autosomal synapsis but have achiasmatic homolog pairs, or rather fail to synapse the X-Y chromosomes, they undergo apoptosis exclusively at epithelial stage XII, when metaphase I spermatocytes experience the first meiotic division (Eaker et al. 2002; Lipkin et al. 2002; Barchi et al. 2008; Kauppi et al. 2013a). We thus reasoned that if the germ cell loss observed in *Tg*(*Spo11*)^+/−^ tubules was due to both a defect in proper synapsis of the autosomes and segregation at metaphase I, we should have observed a significant increase of apoptosis in both stages IV and XII of the epithelial cell cycle. Our analysis revealed that in *Tg*(*Spo11*)^+/−^ mice, there was a significant increase in the proportion of stage IV tubules with at least one apoptotic cell (58.3 ± 17 % *Spo11*^+/−^ vs. 81.6 ± 5 % *Tg*(*Spo11*)^+/−^, *p* = 0.03, *t* test on arcsin-transformed percentages). In addition, as shown in Fig. 2d and quantified in Fig. 2e, the average number of apoptotic cells during stage IV increased significantly from 2.8 ± 0.7 in *Spo11*^+/−^ to 5.9 ± 1.6 in *Tg*(*Spo11*)^+/−^, suggesting that germ cells were lost at the stage IV elimination point (Hamer et al. 2008). In contrast, no significant increase of apoptosis was observed during stage XII (data not shown). Overall, these observations indicated that although spermatogenesis in *Tg*(*Spo11*)^+/−^ mice is substantially normal, a subset of spermatocytes is eliminated by apoptosis, likely due to defects in chromosome synapsis of the autosomes.

### *Tg*(*Spo11*)^+/−^ mice display an overall reduced expression of SPO11

**Fig. 3:**
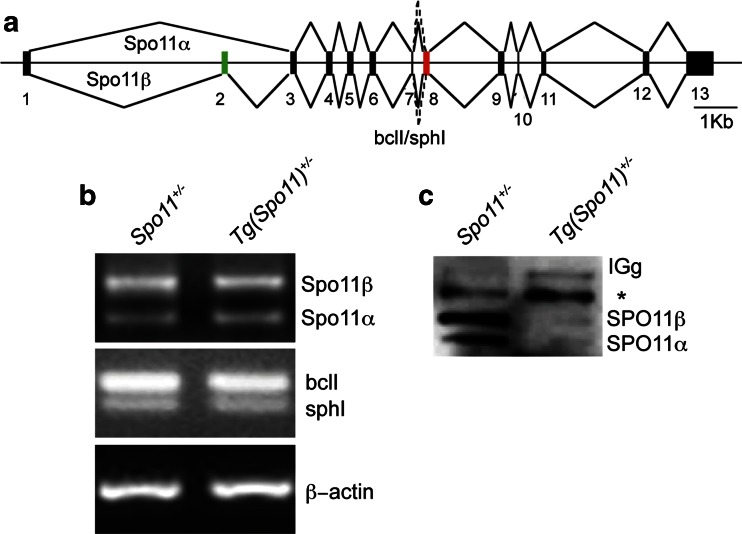
Expression analysis of *Spo11* splicing isoforms. **a** *Spo11* genomic organization and splicing pattern. Splice variant α excludes exon 2, whereas variant β includes it. The splice junction between exons 7 and 8 varies with respect to the site of the 3′ splice acceptor, including (*bclI*) or excluding (*sphI*) 12 nucleotides. **b** Representative RT-PCR analyses of the expression of *Spo11* splicing variants in the indicated genotypes. Actin PCR was used as loading control. **c** Immunoprecipitation of SPO11 in the indicated genotypes. Two independent experiments were performed, one is shown here. The *asterisk* indicates low-mobility bands likely originating from the *Spo11* knockout allele expressed in more advanced cell types as demonstrated earlier (e.g., see Kauppi et al. 2013a and supplemental Fig. 2). Note that in *Tg*(*Spo11*)^+/−^ mice, the low-mobility band is stronger than in *Spo11* ^+/−^ as mice carry two copies of the knockout allele

The establishment of proper synapsis between homologs requires that SPO11 splicing variants (Fig. 3a) are correctly expressed (Kauppi et al. 2011). To understand whether the above-described abnormalities in meiotic progression and apoptosis (Fig. 2) could be linked to the lack of expression of a specific *Spo11*-splicing isoform from the transgene, we amplified the cDNA from 15 days post-partum (dpp) *Tg*(*Spo11*)^+/−^ testis using isoform-specific primers. As shown in Fig. 3b, *Spo11β* and *Spo11α* as well as *Spo11*-*BclI Spo11*-*SphI* transcripts were expressed. In addition, to verify whether the two major isoforms (SPO11β and SPO11α) were properly translated, we immunoprecipitated SPO11 from total testis lysate of 45-dpp-old mice. As shown in Fig. 3c, although both isoforms were present, overall protein expression was reduced with respect to *Spo11*^+/−^ control mice. Since in many organisms proper SPO11 expression and DSB number are critical for correct recombination-dependent homologous synapsis (Davis et al. 2001; Tesse et al. 2003; Henderson and Keeney 2004; Kauppi et al. 2013a, b), we next analyzed whether the observed reduction in SPO11 expression in *Tg*(*Spo11*)^+/−^ mice resulted in any appreciable alteration of chromosome dynamics.

### A large fraction of early/mid-zygonema spermatocytes of *Tg*(*Spo11*)^+/−^ mice display chromosome synapsis defects

In mammals, recombination-driven pairing of homologs and synapsis can be monitored by staining meiotic chromosome spreads with antibodies that recognize the axial/lateral element (AE/LE) and central element (CE) of the SC. The SC is a proteinaceous structure that juxtaposes homologous chromosomes in close proximity during the repair of SPO11-mediated DSBs. The SC comprises several proteins, including the AE/LE protein SYCP3, the assembly of which begins at leptonema. The CE protein SYCP1 assembles along chromosome axes when synapsis of homologous chromosomes commences (Page and Hawley 2004). In normal spermatocytes at leptonema, axial element formation appears as short stretches of SYCP3 staining. When cells progress to early zygonema, the initiation of chromosome synapsis (visible by the assembly of the SYCP1-positive central element of the SC) occurs simultaneously with the elongation of the axial (now referred to as lateral) element. As cells proceed to mid/late zygonema, the elongation of chromosome axes is completed, detectable by uninterrupted SYCP3 staining, and homologous chromosome synapsis is achieved over at least 50 % of their length.

**Fig. 4:**
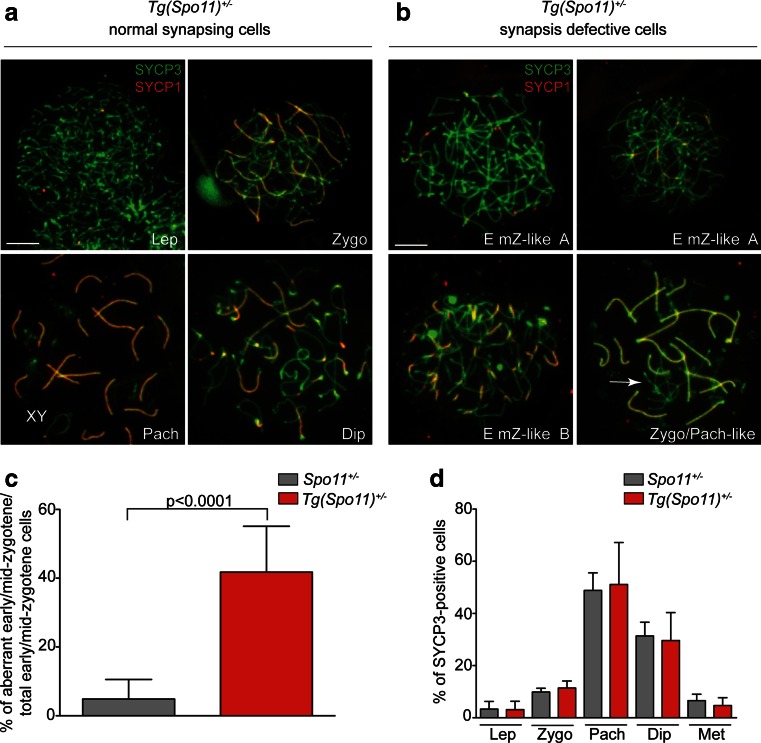
Analysis of chromosome synapsis abnormalities and meiotic cell progression, in *Tg*(*Spo11*)^+/−^ and control spermatocytes. Chromosome spreads of *Tg*(*Spo11*)^+/−^ cells immunolabeled for SYCP3 (*green*) and SYCP1 (*red*). **a** Representative images of normal synapsing cells at the indicated stages of prophase I. *Lep* = leptonema, *Zygo* = zygonema, *Pach* = pachynema, *Dip* = diplonema. *XY* indicates the sex chromosomes. **b** Representative images of early/mid-zygotene-like cells of type A (*Em Z-like A*), type B (*Em Z-like B*), and of a late zygotene/pachytene-like (*Zygo/Pach-like*) cell. The latter cell type was defined as a cell where chromosome synapsis was extended over 50 % and one or more fully elongated (mature) axes (identified by SYCP3 staining) remained completely unsynapsed (*white arrow*) indicating asynchrony with respect to bulk chromosomes. *Magnification bar* represents 5 μm. **c** Quantification of early/mid-zygotene-like cells within the early/mid-zygonema class, in the indicated genotypes [*Spo11* ^+/−^ *, n* = 137 nuclei analyzed; *Tg*(*Spo11*)^+/−^, *n* = 196 nuclei analyzed; six mice tested for each genotype], *t* test on arcsin-transformed percentages. *Error bars* = mean ± SD. **d** Spermatocytes from 45 to 93 dpp mice of the indicated genotypes were stained for SYCP1/SYCP3 and categorized accordingly to meiotic stage. The zygotene class in *Tg*(*Spo11*)^+/−^ spermatocytes includes both normally and abnormally synapsing cells [*Spo11* ^+/−^, *n* = 319 nuclei; *Tg*(*Spo11*)^+/−^, *n* = 335 nuclei, three mice tested for each genotype, Bonferroni test, *p* = 0.7]. *Error bars* = mean ± SD. *Met* = metaphase I and metaphase II cells

**Fig. 5:**
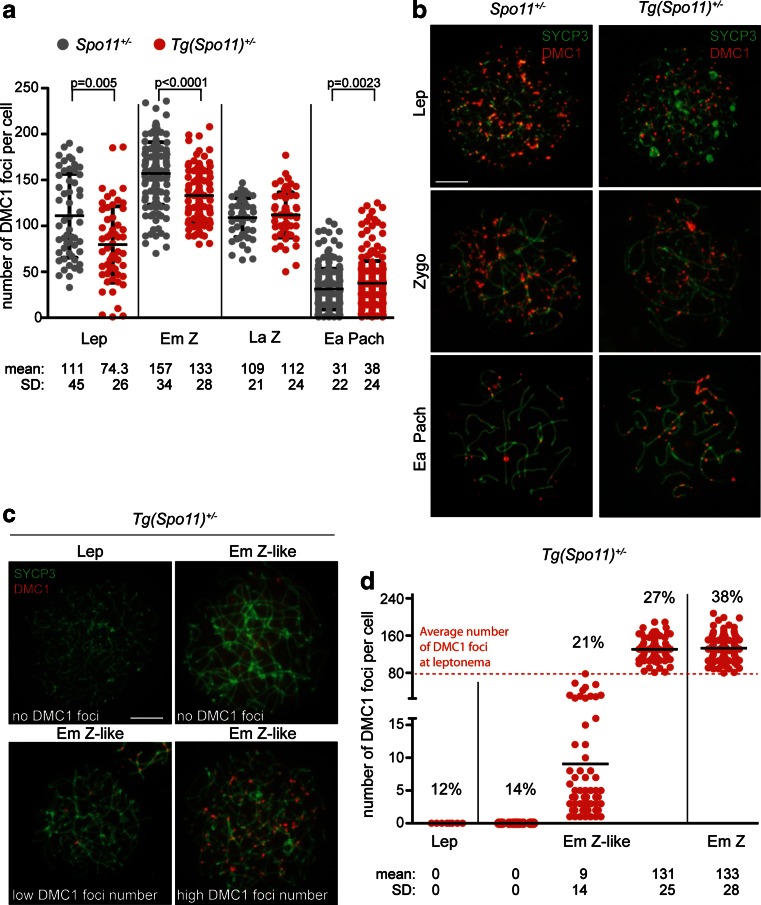
*Tg*(*Spo11*)^+/−^ spermatocytes undergo a reduced/delayed wave of DSBs. **a** Quantification of DMC1 foci number in the indicated genotypes. In *Tg*(*Spo11*)^+/−^ mice, DMC1 foci number is decreased significantly at leptonema (*Lep*) [*Spo11* ^+/−^, *n* = 54 nuclei, *Tg*(*Spo11*)^+/−^. *n* = 59 nuclei] and normal early/mid-zygonema (*Em Z*) [*Spo11* ^+/−^, *n* = 142 nuclei, *Tg*(*Spo11*)^+/−^, *n* = 113 nuclei], while it is increased at early pachytene stage (*Ea Pach*) [*Spo11* ^+/−^, *n* = 209 nuclei; *Tg*(*Spo11*)^+/−^, *n* = 262 nuclei]; six mice tested for each genotype. **b** Representative nuclear spreads, from the indicated genotypes, labeled with anti-DMC1 and anti-SYCP3. *Scale bar* 5 μm. **c** Representative images of leptotene (*Lep*) and early/mid-zygotene-like (Em Z-like) nuclei with no DMC1 foci, and Em Z-like cells with high and low number of DMC1 foci. *Scale bar* 5 μm. **d** *Numbers in parentheses* are quantification of nuclei with no DMC1 foci at leptotene and early/mid-zygotene-like stages and nuclei with DMC1 foci in normal and aberrant early/mid-zygotene cells of *Tg*(*Spo11*)^+/−^ mice (leptotene stage, *n* = 62; early/mid-zygotene, *n* = 209). Each *dot* in the graph indicates the number of DMC1 foci per nucleus, in normal and Em Z-like cells of *Tg*(*Spo11*)^+/−^. The *dotted line* indicates the average number of DSBs at leptonema, in *Tg*(*Spo11*)^+/−^ mice. *Black bars* are means and standard deviations, *p* = *p* values (one-tailed Mann-Whitney test)

Analysis of chromosome synapsis in *Tg*(*Spo11*)^+/−^ mice revealed that when cells progressed past leptonema, normal synapsing cells (Fig. 4a) appeared concomitantly with cells showing abnormal synaptic features. In contrast, autosomal synapsis was normal in males expressing the *Spo11*-IRES-*Cre* transgene in a *Spo11*^+/+^ background (data not shown). Hence, it seems likely that *Tg*(*Spo11*)^+/−^ synaptic defects are tied to the altered expression of the exogenous SPO11 (Fig. 3c), beside other features of the BAC-derived transgene (that is, the expression of *Cre*-recombinase). In accordance with the uninterrupted extension of the SYCP3-positive axial element and the appearance of (SYCP1-positive) chromosome synapsis, most aberrant-looking nuclei were classified as early/mid-zygotene-like (Em Z-like) cells. These aberrant cells which were morphologically similar to class I cells (Kauppi et al. 2013a) were defined as nuclei with fully elongated SYCP3-positive axes (typical of mid-zygonema cells in wild type), with no or very limited overall synapsis (Em Z-like A cells, Figs. 4b and 5c); or nuclei with more extended overall chromosome synapsis (up to 50 %), where multiple fully elongated axes remained unsynapsed (Em Z-like B cells, Fig. 4b) but without obvious signs of nonhomologous synapsis. These aberrant cells are in contrast to normal zygotene cells, in which SYCP3-stained axes elongate concomitantly with homologous synapsis progression (Kauppi et al. 2013a). Since Em Z-like cells were never observed in wild type (data not shown), we viewed this as an indication of defects in synaptic progression. The percentage of normal-looking and aberrant nuclei was quantified in 45–93-dpp-old mice by staining of chromosome spreads with SYCP3/SYCP1 markers. Surprisingly, in *Tg*(*Spo11*)^+/−^ mice, a large fraction (46.3 ± 10.3 %) of early/mid-zygonema cells appeared abnormal. Among Em Z-like cells, the most common single cell type was type A cells (67.3 ± 22 %). Em Z-like cells were never found in wild-type animals, while they were present at a low percentage in *Spo11*^+/−^ mice (4.8 ± 5.4 %; Fig. 4c). Remarkably, when (rare) synaptic-defective cells were found at late zygotene/pachytene-like stage, contrary to other mouse models with reduced SPO11β^bclI^ expression (Kauppi et al. 2011, 2013b), chromosome tangles were rarely apparent (Fig. 4b), suggesting that this phenomenon was somehow prevented.

Spermatogenesis is a continuous process in which spermatocytes mature into spermatids by passing through meiotic sub-stages. If abnormal synapsis of *Tg*(*Spo11*)^+/−^ spermatocytes remained unresolved, cells die at the stage IV elimination point (de Rooij and de Boer 2003; Barchi et al. 2005; Hamer et al. 2008), with consequent depletion of post-zygotene cell types. However, as quantified in Fig. 2e, in *Tg*(*Spo11*)^+/−^ testis, apoptosis increased only modestly. In addition, the analysis of meiotic cells using prophase I chromosomes stained for SYCP3/SYCP1 markers revealed that the frequency of post-zygotene nuclei of *Tg*(*Spo11*)^+/−^ mice was not significantly perturbed compared to that of *Spo11*^+/−^ mice (Fig. 4d), indicating that no significant loss of germ cells at zygonema had occurred. Overall, these observations suggest that most of the cells with aberrant synapsis morphology likely resolved their synaptic problems before reaching the stage IV elimination point and subsequently progressed through prophase I unperturbed.

In mice, as in other species, timely formation, and repair, of an appropriate number of DSBs is critical for proper pairing and synapsis of the homologs (Davis et al. 2001; Tesse et al. 2003; Henderson and Keeney 2004; Kauppi et al. 2013a). To get an insight into the mechanism behind the apparent phenotypic rescue observed in *Tg*(*Spo11*)^+/−^ mice, we next analyzed DSB formation and resolution during prophase I.

### Spermatocytes of *Tg*(*Spo11*)^+/−^ mice receive a reduced/late wave of DSBs

Timing and levels of DSBs can be studied by staining chromosomal spreads with antibodies against the AE/LE element of the SC component SYCP3, and DMC1, a protein known to bind to single strand DNA tails of DSBs (Bishop 1994; Keeney 2001). In agreement with the results above, by analyzing SYCP3-stained spreads of adult spermatocytes, we found that in *Tg*(*Spo11*)^+/−^ mice, both normal and Em Z-like spermatocytes were present, with the latter being the most abundant (61 ± 9 %, *n* = 322) among early/mid-zygotene cells. The analysis of DMC1 foci deposition in *Tg*(*Spo11*)^+/−^ mice at leptonema, and in normal early/mid-zygotene cells, revealed that consistent with the observed reduction in SPO11 protein level (Fig. 3c), the average number of DMC1 foci was reduced to 33 and 20 %, respectively, compared to in *Spo11*^+/−^ mice, Fig. 5a, b [~58 and ~40 % of wild-type level (see supplemental Table 2)]. The first semi-synchronous wave in 15 dpp juvenile mice yielded a result comparable with that in adults (Supplemental Fig. 3). However, in *Tg*(*Spo11*)^+/−^ spermatocytes at early pachynema, the average number of DMC1 foci was significantly increased compared to the controls (Fig. 5a, b), indicating that in these mice, either meiotic DSB repair was delayed or a fraction of pachytene cells developed from zygotene stage cells that had received a delayed wave of DSBs. Since in *Tg*(*Spo11*)^+/−^ spermatocytes the proficiency of DSBs repair is not expected to be significantly altered with respect to *Spo11*^+/−^ cells, we favor the latter interpretation.

The quantification of DMC1 foci number in Em Z-like cells revealed that 14 % had no detectable foci. Interestingly, this percentage closely matched that of leptotene stage cells with no foci (12 %), possibly indicating that most Em Z-like cells with no DMC1 foci had never received DSBs (Fig. 5c, d). Nevertheless, the vast majority of Em Z-like cells displayed DMC1 foci, although with highly variable numbers (Fig. 5d). As shown in Fig. 5a, most normal early/mid-zygonema cells of *Tg*(*Spo11*)^+/−^ mice displayed a number of DSBs that is higher than the average number of DMC1 foci at leptonema. Because these cells are expected to progress normally through prophase I, such a level of DSBs is likely above the threshold level that supports normal chromosome synapsis (Kauppi et al. 2013a, b). In order to estimate the percentage of Em Z-like cells that receive a surge of DSBs that promote homologous synapsis, we divided Em Z-like cells with foci in two groups: those with DMC1 foci count above and below the average number of foci at leptonema. As shown in Fig. 5c, with this analysis, we estimated that among early/mid-zygotene nuclei, 27 % were Em Z-like cells with an average number of DMC1 foci indistinguishable with respect to that of early/mid-zygotene nuclei with no synaptic defects (*p* = 0.6, two-tailed Mann-Whitney test), providing a mechanistic link for their rescue. In the remaining fraction of Em Z-like cells, which account for 21 % of the cells at early/mid-zygonema, the number of DMC1 foci was instead markedly reduced in most cells (Fig. 5d). Because the extension of chromosome synapsis in this latter group was similar, in most spermatocytes, to that of cells with high DMC1 foci count (Fig. 5b and supplemental Fig. 4), one possible interpretation is that Em Z-like cells with high number of DMC1 foci developed from cells with low foci number, following a rapid surge of DSBs. However, additional mechanisms of rescue cannot be excluded (see “Discussion”).

**Fig. 6:**
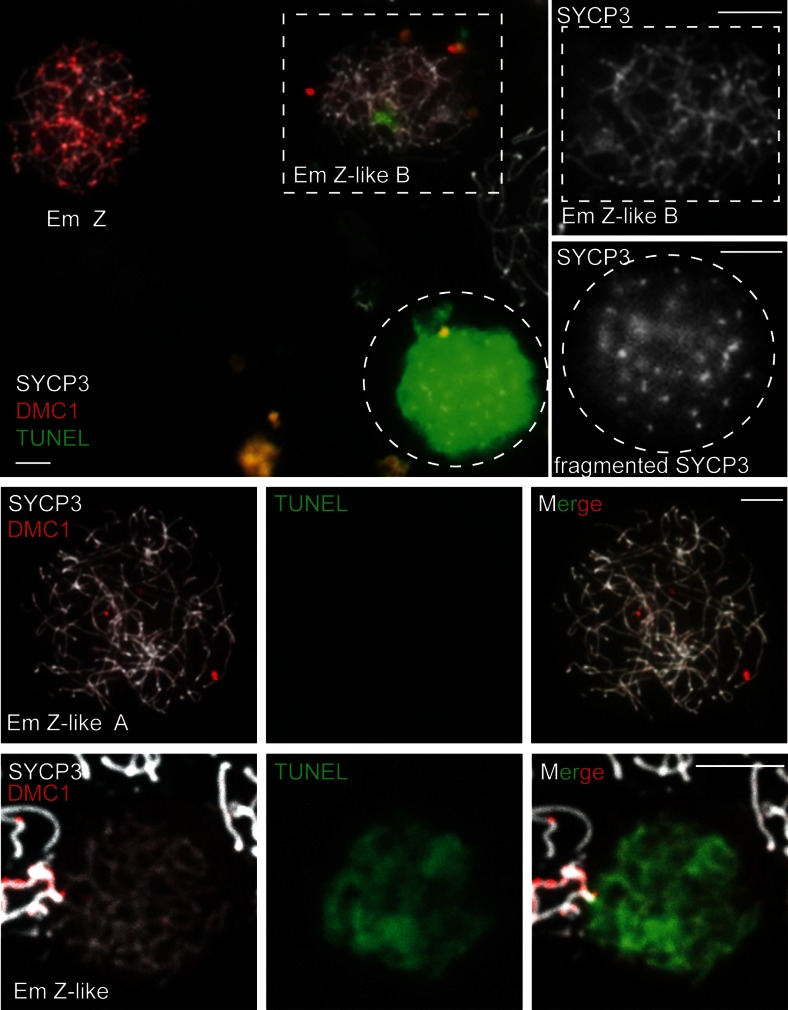
Em Z-like cells with no DMC1 foci die by apoptosis. Representative images of cells from *Tg*(*Spo11*)^+/−^ mice, stained with SYCP3, DMC1, and TUNEL. *Top row*: TUNEL-negative early/mid-zygotene-like B (*Em Z-like B*) cell with no foci (highlighted in the *dotted square*) and a normal early/mid-zygotene (*E mz*) cell with DMC1 foci, compared to a TUNEL-positive nucleus (Em Z-like B, *dotted circle*). Images on the *right* are magnifications of the highlighted cells, showing the morphology of the SYCP3-stained axes. *Mid row*: representative image of an Em Z-like A cell with no TUNEL signal, indicative of the fact that Em Z-like cells persist before being eliminated. *Bottom row*: representative image of a rare TUNEL-positive Em Z-like cell with no DMC1 foci, where SYCP3-stained axes appeared still intact. *Scale bar* 5 μm

In addition to an appropriate level of DSBs, the rescue of Em Z-like cells would only occur if the DSBs were received before cells undergo apoptosis. To understand if Em Z-like nuclei were apoptotic, we performed a terminal deoxynucleotidyl transferase dUTP nick end labeling (TUNEL) assay on cells stained for DMC1/SYCP3. We focused our analysis on cells with no DMC1 foci, as they were the ones that most likely would be eliminated. We observed that most TUNEL-positive cells were negative for both DMC1 and SYCP3, precluding the analysis (not shown). However, the rare TUNEL-positive spermatocytes where SYCP3-positive axes were still maintained (*n* = 23) lacked DMC1 foci (Fig. 6). Assuming that in TUNEL-positive nuclei, the DMC1 marker is not lost precociously, this observation suggests that the majority of cells in which DSBs were made survived. Interestingly, we also noticed that out of 14 Em Z-like cells with no DMC1 foci and well-preserved SYCP3, none were TUNEL-positive (Fig. 6). This indicates that Em Z-like cells persist for a certain time, before being eliminated.

Overall, these results indicate that normal synapsing cells of *Tg*(*Spo11*)^+/−^ mice receive, by early/mid-zygonema, a reduced (above threshold level) number of DSBs. However, DSBs do not form timely in all spermatocytes, leading to the formation of Em Z-like cells. The latter last within a time window that precedes their apoptotic elimination and are rescued by a late surge of DSBs.

## Discussion

Over recent years, the complementation analysis of the *Spo11*^−/−^ phenotype by different *Spo11*-expressing transgenes turned out to be an excellent tool for understanding the requirements and dynamics of homologous chromosome pairing in meiosis. The complementation analysis of *Spo11* knockout by a transgene that expresses at least wild-type levels of SPO11β^bclI^ under the control of the *Xmr* promoter has revealed that while the expression of this splicing isoform is sufficient to rescue autosome synapsis, it does not support synapsis of XY chromosomes (Kauppi et al. 2011). In addition to SPO11β, SPO11α is the other main SPO11 splicing isoform (Keeney et al. 1999; Romanienko and Camerini-Otero 1999; Bellani et al. 2010); therefore, it has been proposed that SPO11α is required to promote sex chromosome segregation in males (Kauppi et al. 2011). In addition to the above mouse model, it was demonstrated that in mice, as in other species in which recombination is required for stable pairing (e.g., see Terasawa et al. 1995; Lenzi et al. 2005; Storlazzi et al. 2010), the number of SPO11-generated DSBs also matters. Indeed, when DSBs generated by SPO11β were critically reduced with respect to wild type (~50 % at leptonema, ~75 % at early/mid-zygonema [see supplemental Table 2]) along with XY chromosomes, autosome synapsis also failed, and spermatocytes with entangled chromosomes were eliminated at a pachytene-like stage, by apoptosis (Kauppi et al. 2013a). In contrast, meiotic progression of *Spo11* heterozygous mice is completely normal (Bellani et al. 2005; Cole et al. 2012; Kauppi et al. 2013a). Whether *Spo11*^+/−^ DSB levels represent or approximate the lower threshold lower limit that can still support chromosome synapsis is yet unknown. In addition, it is worth noting that because in the mouse model of Kauppi et al. (2013a) a reduction in DSB levels was obtained by expressing only the SPO11β^bclI^ isoform, it could not be excluded that the lack of a full suite of *Spo11* splicing isoforms makes spermatocytes somehow more prone to entanglement of their chromosomes and apoptosis (Kauppi et al. 2013a) (see below).

To study chromosome dynamics in spermatocytes where the ectopic expression of the full set of SPO11 splicing isoforms is not compromised, we complemented *Spo11* deletion using the *Spo11*-IRES-*Cre* transgene. Our analyses revealed that in *Tg*(*Spo11*)^+/−^ mice, overall SPO11 expression was remarkably reduced with respect to *Spo11*^+/−^. Nevertheless, male mice were fully fertile, suggesting that the reduced expression of the protein did not significantly impact sperm production. A similar mechanism may operate in females where the same transgene also results in normal fertility. In agreement with this observation, only a small fraction of testis cross sections displayed abnormalities and few spermatocytes died by apoptosis. An unexpected finding was that despite a histologically normal spermatogenesis, the analysis of surface spreads revealed that a substantial percentage of early/mid-zygotene cells suffered from a delay in chromosome synapsis (Em Z-like cells). During spermatogenesis, timely repair of DSBs and chromosome synapsis are monitored by the prophase I checkpoint. Meiocytes displaying persistent chromosome pairing/synapsis defects are eliminated by apoptosis (de Rooij and de Boer 2003; Barchi et al. 2005; Hamer et al. 2008; Mahadevaiah et al. 2008), with consequent depletion of the germ cell population. The lack of any relevant reduction of post-zygotene cell populations in our model suggested that synapsis abnormalities of most Em Z-like cells were only present transiently and were likely resolved before their possible elimination at the checkpoint.

To gain insight into the mechanism behind this rescue, we analyzed DSB numbers and dynamics, counting DMC1 foci deposition during different meiotic sub-stages. The quantification of DMC1 foci in leptotene spermatocytes from *Tg*(*Spo11*)^+/−^, revealed that DSB numbers were reduced by 33 % compared to those in *Spo11*^+/−^ mice (~58 % with respect to wild type [supplemental Table 2]). In addition, a small percentage of spermatocytes did not have foci (see below). A very similar reduction in DMC1 foci number was previously found in leptotene spermatocytes expressing a reduced dosage of SPO11β^bclI^ on a *Spo11*^−/−^ background [see (Kauppi et al. 2013a) and supplemental Table 2]. This indicates that in both models, the reduced expression of SPO11 decreases DSB numbers at the onset of meiosis. Quantification of DMC1 foci number in normally synapsing cells of *Tg*(*Spo11*)^+/−^ spermatocytes at zygonema (Fig. 5a) showed that although the level of DSBs increased during the leptonema to early/mid-zygonema transition, their absolute number was overall reduced in respect to *Spo11*^+/−^ mice. This observation sets the absolute lower limit of DSB numbers for successful chromosome synapsis at this stage, below that of *Spo11*^+/−^ spermatocytes [approximately in the range of 0.25–0.6 of the wild-type level (supplemental Table 2)]. The reduction of DMC1 foci number at early/mid-zygonema in *Tg*(*Spo11*)^+/−^ may be the result of fewer DSBs, consistent with the level of SPO11 protein expression. However, an alternative to reduced absolute levels of DSBs is that spermatocytes have a modified kinetic of DSB formation. In support of this interpretation, DMC1 foci number at early pachynema were significantly increased compared to those in *Spo11*^+/−^. Since DSB formation on unsynapsed axes is a continuous process (Kauppi et al. 2013a), a likely scenario is that during early zygonema, in *Spo11*^+/−^ spermatocytes, the rate of DSB formation overcomes that of repair (surge). When cells progress toward late zygonema, DSB formation rate is reduced, causing a stiff reduction of overall DMC1 foci number. Conversely, in *Tg*(*Spo11*)^+/−^ cells, the rate of DSBs formation is reduced at leptonema and early zygonema, with respect to *Spo11*^+/−^; however, new DSBs continue to be formed by late zygonema with higher frequency than the control and are therefore present in a greater number by early pachynema. Recent evidence implicates ATM kinase in regulating SPO11 DSB formation in mouse spermatocytes through a negative feedback loop (Lange et al. 2011). It is thus likely that, in *Tg*(*Spo11*)^+/−^ spermatocytes, early reduced DSB formation at leptonema yields a reduced ATM activation and thereby makes cells responsive to mechanisms that are consistent with SPO11 expression levels sustain SPO11 activity at early/mid-zygonema and late zygonema. However, one argument against the hypothesis that in *Tg*(*Spo11*)^+/−^ cells a reduced load of DSBs (and ATM activation) promotes the surge at early/mid-zygonema, is that it does not occur when low levels of SPO11β^bclI^ are expressed on a *Spo11*^−/−^ background (Kauppi et al. 2013a). Because the latter model lacks the full complement of *Spo11* splicing isoforms, one possibility is that, in mice, SPO11 activity is subjected to both a negative (ATM-dependent) and positive controls and that the absence of some of the splicing isoforms makes cells unresponsive to mechanisms that positively regulate DSB numbers (surge). Since Mec1 [ataxia telangiectasia-mutated and Rad3-related (ATR) in mammals] has recently been implicated in a positive feedback loop that regulates DSB numbers in yeast (Argunhan et al. 2013; Gray et al. 2013; Cooper et al. 2014), we speculated that SPO11 splicing isoforms (and/or their not yet identified interacting partners) might be targeted by the ATR kinase. If correct, this scenario suggests a multi-layered control of DSB formation in which ATM and ATR, interacting with SPO11 and/or its functional partners, play opposite regulatory roles to guarantee the homeostatic control of DSB formation, under sub-optimal SPO11 expression conditions. Importantly, also in wild-type cells, recombination initiation is a stochastic process, in which at leptonema, meiotic cell receives a widely variable number of DSBs, potentially creating the basis for the establishment of chromosome synapsis defects. Variations in DSB number or kinetics would thus operate also in wild type, on a cell-to-cell basis, depending on the initial load of DSBs and perhaps variability in expression timing of SPO11.

As reported above, along with cells whose chromosome synapsis at zygonema appeared normal, in *Tg*(*Spo11*)^+/−^ spermatocytes, a substantial fraction of early/mid-zygotene cells displayed features of a delay in chromosome synapsis. However, cells with nonhomologous synapsis were rarely apparent, indicating that, as also suggested by others, in mice, nonhomologous synapsis at zygonema is somehow disfavored [see (Kauppi et al. 2013a) and references therein]. Interestingly, however, contrary to what was observed in the model of Kauppi et al. (2013a), such cells never arrested at a pachynema-like stage. Chromosome synapsis defects are thus likely resolved before this arrest point. One possible scenario is that Em Z-like cells of *Tg*(*Spo11*)^+/−^ mice received a late but “above threshold level” surge of DSBs, reducing the chance that orphan chromosomes (often the X chromosome) become engaged in nonhomologous interactions [see the model proposed in (Kauppi et al. 2013a)]. Indeed, in support of this interpretation, we found that a relevant fraction of Em Z-like cells (~27 % of total early/mid-zygonema nuclei) received a surge of DSBs identical to that of normal-progressing cells at early/mid-zygotene (Fig. 5d). However, not all Em Z-like cells behaved similarly, as a small group of cells (~14 % of early/mid-zygotene stage cells) did not display DMC1 foci. Their percentage almost matched that of leptotene cells with no foci, suggesting that in this subpopulation, SPO11 was either not expressed or expressed at a negligible level, possibly due to a defective expression of the transgene. As indicated by TUNEL assay, these cells likely die by apoptosis at stage IV. In a third group of Em Z-like spermatocytes (~21 % of cells at early/mid-zygonema), we observed a very wide variation in Dmc1 foci number (from 1 to 79) with most cells displaying 1–15 foci. The latter possibly die or are rescued. The outcome might depend on whether they receive an “above threshold level” number of DSBs in a timely fashion, in order to guarantee stable synapsis between the homologs. The small increase in apoptosis observed histologically results from TUNEL assay and the lack of a significant depletion of post-zygonema population in *Tg*(*Spo11*)^+/−^ mice, suggesting that most of these cells will likely complete synapsis before reaching the stage IV elimination point. Intriguingly, recent findings have demonstrated that in yeast, during prophase I, the fate of late-forming DSB changes over time, with late-forming DSBs showing a stronger homologous bias than early ones (Joshi et al. 2015). This is similar to the predicted role, in mice, of SPO11α in promoting late-forming DSBs, which are more prone to contribute to homologous recognition and segregation of the sex chromosomes (Kauppi et al. 2011). In accordance with these findings, in addition to the formation of a set number of DSBs that overcomes the threshold level, possibly, in some Em Z-like cells, relatively low numbers of DSBs are made late and are more prone to contribute to correct chromosome synapsis, thus being sufficient to rescue cells before the apoptotic elimination point. In this perspective, one could imagine that the threshold would tend to decline over time. The outcome, however, might depend on the overall status of synapsis at the time when late-forming DSBs are made, with cells with more advanced synapsis (that is, Em Z-like type B cells), being perhaps more prone to be rescued by this latter mechanism.

**Fig. 7:**
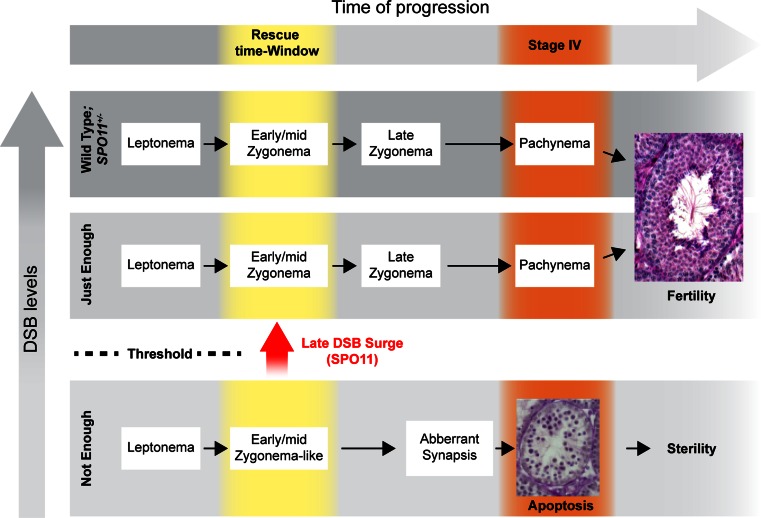
Model summarizing the effect of DSB reduction and late DSB formation on spermatocyte meiotic progression. In wild-type and *Spo11* ^+/−^ mice, double-strand break (DSB) levels support proper chromosome synapsis and meiotic progression (*top gray horizontal bar*) and spermatocyte progress through prophase I, up to the formation of mature sperms. If DSBs are reduced below the level of *Spo11* ^+/−^, but above the threshold (just enough), spermatogenesis is successful (*mid gray horizontal bar*). However, if at leptonema and early/mid-zygonema, “not enough” DSBs are generated (*bottom gray horizontal bar*), chromosome synapsis is delayed, and early/mid-zygotene-like spermatocytes appear. If no (or below-threshold levels) DSBs are further generated, spermatocytes undergo nonhomologous synapsis, arrest at a pachytene-like stage, and die by apoptosis at stage IV of the epithelial cell cycle (Kauppi et al. 2013a). In contrast, if early/mid-zygotene-like cells receive a late surge of DSBs before chromosomes entangle and start to die (rescue time window), chromosome synapsis is rescued and cells progress through prophase I, up to the formation of mature sperms

In conclusion, in *Tg*(*Spo11*)^+/−^ mice, cells are able to cope with reduced (below heterozygosity) levels of DSBs. Likely, this is due to the presence of the full set of *Spo11* splicing isoforms that, even when SPO11 expression was reduced, allow cells to still receive a surge of DSBs at zygonema that overcome the threshold level, promoting proper homologous search. However, when cells at leptonema and early/mid-zygonema receive too few DSBs, chromosome synapsis initiation is delayed and cells reach an Em Z-like stage without evident signs of nonhomologous synapsis. These cells persist within a time window where nonhomologous synapsis is prevented, and they are competent to receive a late surge of DSBs. If late-forming DSBs are above “threshold level” spermatocytes, synapsis progresses further and is completed successfully, leading to an overall normal progression of cells through the successive phases of spermatogenesis (summarized in Fig. 7).

## Material and methods

### Generation of *Tg*(*Spo11*)^+/−^ mice and transgene copy number

*Spo11*^−/−^ [*TgSpo11*-IRES-*Cre*] mice, named *Tg*(*Spo11*)^+/−^, were obtained at first, by crossing mice carrying Tg*Spo11*-IRES-*Cre* transgene in a wild-type background (Pellegrini et al. 2011), with *Spo11*^+/−^ mice (Baudat et al. 2000). Then, the resulting *Spo11*^+/−^ [*TgSpo11*-IRES-*Cre*] offspring was crossed again with *Spo11*^+/−^ mates. All mice were maintained in a C57/BL6 X 129S/v mix background. To minimize variability from strain background, experimental animals were compared to controls from the same litter or from the same mating involving closely related parents. Each analysis was done with at least three animals.

In order to quantify Tg(*Spo11*-IRES-*Cre*) transgene copy number in *Tg*(*Spo11*)^+/−^ mice, we performed a real-time PCR assay, based on evaluation of genomic *Spo11* sequence from the transgene. Probe and primers were specific for exon 5 of the *Spo11* gene, replaced by the *Neo* cassette in the knockout allele (Baudat et al. 2000). Multiple generations from *Tg*(*Spo11*)^+/−^ mice (*n* = 4) were tested to exclude any recombination event and changes in transgene copy number through successive generation. All transgenic mice analyzed resulted to be in concordance with *Spo11* wild-type copy number (two copies), used as relative control, while one copy was obtained from *Spo11*^+/−^, as expected. No *Spo11* sequence amplification was observed with DNA from *Spo11*^−/−^ mice (negative control).

*Tg*(*Spo11*)^+/−^ mice generated close to 50 % of transgenic and 50 % of non-transgenic progeny and copy number were similar among transgenic littermates, consistent with a single stable site of *Spo11*-IRES-*Cre* BAC transgene integration in the founder. Thus, *Tg*(*Spo11*)^+/−^ are hemizygous for Spo11-IRES-Cre transgene integrated in two copies array at a single stable site. Real-time PCR was performed using a TaqMan-based assay (Life Technologies) with Taqman Universal master mix II (Life Technologies). The comparative ΔΔCt method was used to quantify relative gene expression. Primers and probes for *Spo11* gene were designed as follows: *Spo11* probe 5′-6 FAM-TATGCTGAAAGTGCCCAGG-MGB-3′; primers: *Spo11* sense 5′-CGCCATCGATGACATTTCC-3′; and *Spo11* antisense 5′-GGCTCACCACGTGCAGACT-3′. The expression of mouse *ApoB* was used as reference gene; *ApoB* Probe VIC 5′-CCA ATG GTC GGG CAC TGC TCA A-3′ MGB, Primers ApoB sense 5′-CAC GTG GGC TCC AGC ATT-3′, and ApoB antisense 5′-TCACCAGTCATTTCTGCCTTT-3′. All primers and probes for real-time PCR assay were designed with Primer Express Software v3.0.1 (Life Technologies, Applied Biosystems).

### Genotyping

Genotyping was performed by both conventional and real-time PCR of tail tip DNA. Genotyping of *Cre* sequence was executed with conventional PCR by using the following set of primers: Cre sense 5′-AAT GCT TCT GTC CGT TTG CCG G-3′ and Cre antisense 5′-CCA GGC TAA GTG CCT TCT CTAC-3′. Genotyping of *Spo11* endogenous allele was performed by conventional PCR by using the following primers: Sp1F sense 5′-CTA CCT AGA TTC TGG TCT AAG C-3′, PRSF2 5′-CTG AGC CCA GAA AGC GAA GGA A-3′, and Sp16R 5′-ATG TTA GTC GGC ACA GCA GTA G-3′ (Baudat et al. 2000). Since PCR primers Sp1F/Sp16R also recognize the *Spo11* wild-type allele from the BAC vector, to identify mice carrying the transgene within an endogenous *knockout* genetic background [*Tg*(*Spo11*)^+/−^ mice], we determined the *Neomicine* (Neo) cassette copy number from the *knockout* allele (Baudat et al. 2000). Indeed, the presence of two copies of the Neo cassette identified the *Spo11*^−/−^ mice, implying that *Spo11* conventional PCR positivity was due to the transgenic copy. To quantitate Neo copy number, we used a TaqMan Real-Time PCR (Life Technologies) assay, performed as previously described and *ApoB* was used as reference gene. Primers and probes were designed with Primer Express Software v3.0.1 (Life Technologies, Applied Biosystems): *Neo* Probe FAM 5′-TGGCCGCTTTTCT-3′ MGB and primers Neo sense 5′-TGC CGA ATA TCA TGG TGG AA-3′ and Neo antisense 5′-GAT TCA TCG ACT GTG GCCG-3′.

### Tissue collection and histological analyses

Testes and epididymis were collected and fixed overnight at 4 °C in Bouin’s fixative (Sigma no. HT10132). Freshly collected testes were weighted before fixation. Mean of testis weights was calculated and normalized on body weight to minimize difference among animals. Fixed and paraffin-embedded testes were sectioned at 5 μm and stained with periodic acid-Schiff/hematoxylin. For histological evaluation of tubule abnormalities and cell death, cross sections were collected at 100 μm distance. Staging of spermatogenesis was performed according to the method of Russell et al. (1990) and Ahmed and de Rooij (2009). Paraffin-embedded epididymes were stained with Hoechst. Coverslips were mounted using ProLong® Gold Antifade Mountant (Molecular Probes no. P36934).

### Surface chromosome spread preparation and immunofluorescence

Prophase I chromosome preparations and immunofluorescence were performed using previously described techniques (Barchi et al. 2005, 2009). In brief, testes were removed from euthanized animal, decapsulated, chopped in MEM-HG, and mixed. Suspension was let to settle down and supernatant was spun down at 7,500 rpm for 1 min. The pellet was resuspended in 0.5 M sucrose. The suspension was added to slides coated with PFA 1% and 0.015 % TX-100 and incubated for 2 h in a humidified chamber at room temperature. At the end of incubation, slides were rinsed twice in 1:250 Photo-flo Kodak professional (no. 1464510) in water and allowed to air dry. Surface chromosome spreads were either immediately processed for immunofluorescence or stored at –80 °C for up to 6 months. For immunofluorescence, surface chromosome spread preparations were incubated overnight at room temperature with the primary antibody diluted in antibody dilution buffer (ADB, 10 % goat serum, 3 % bovine serum albumin [BSA], 0.05 % Triton X-100 in phosphate-buffered saline [PBS]). At the end of the incubation, slides were washed once in washing buffer 1 (0.4 % Photo-flo, 0.01 % Triton X-100 in water) and once in washing buffer 2 (0.4 % Kodak Photo-flo in water) for 10 min. Slides were incubated with the secondary antibody for 1 h in a pre-warmed humidified chamber at 37 °C in the dark. After 10-min washes with washing buffers 1 and 2, slides were immersed for 1 min into 1× PBS and dried at room temperature in the dark. Coverslips were mounted using ProLong® Gold Antifade Mountant with DAPI (Molecular Probes no. P36935). Images were captured using Leica CTR6000 Digital Inverted Microscope connected to a charge-coupled device camera and analyzed using the Leica software LAS-AF, for fluorescent microscopy. Sources and dilutions of antibodies for immunofluorescence were as follows: mouse anti-SYCP3 (Santa Cruz sc-74569) 1:200, rabbit anti-SYCP1 (Abcam ab15090) 1:200, and rabbit anti-DMC1 (Santacruz no. sc-22768) 1:100. The specificity of anti-DMC1 antibody was verified by staining chromosome spread preparation from *Dmc1*^−/−^ mice. Secondary antibodies used were goat anti-mouse Alexa 488 (no. A1101), goat anti-rabbit Alexa 594 (no. A11012), and goat anti-mouse Alexa 647 (no. 150115) all 1:200, Invitrogen. TUNEL assay was performed according to the method of Pacheco et al. (2015) using the In Situ Cell Death Detection Kit (POD) from Roche.

### RNA extraction and RT-PCR

For RT-PCR, 15-dpp-old mice testes from *Spo11*^+/−^ and *Tg*(*Spo11*)^+/−^ mice were extracted using reagent QIAzol lysis reagent (Qiagen no. 79306). Contaminating genome DNA was removed with DNA-free kit (Ambion no. AM1906). cDNA synthesis was performed with Invitrogen RT-PCR SuperScript III (Invitrogen, no. 18080-051). Spo11β and Spo11α transcripts were amplified with the following primers: Spo11β/α 2F 5′-GTT GGC CAT GGT GAA GAG AG-3′; and Spo11β/α R3 5′-TTT TGG TGA ATC GCT TCT GA-3′. *Spo11* BclI and SphI isoforms were amplified with the following primers: Spo11b/sF 5′-GCCGACTAACATTCAAGGAATGC-3′ and Spo11 b/s R 5′-TGCAGAAGTTGTCGTCCAGGAG-3′.

### SPO11 immunoprecipitation and western blot

Immunoprecipitation (IP) was performed using previously described techniques (Neale et al. 2005; Lange et al. 2011). In brief, testes from 45-dpp *Spo11*^+/−^, *Tg*(*Spo11*)^+/−^, and *Spo11*^−/−^ mice were decapsulated and lysed in 800 μl lysis buffer (1 % Triton X-100, 400 mM NaCl, 25 mM HEPES-NaOH at pH 7.4, 5 mM EDTA). Lysates were subjected to two rounds of centrifugation at 13,200 rpm for 15 min each at 4 °C in a benchtop centrifuge. Supernatants were incubated with monoclonal mouse anti-SPO11 antibody 180 (3 μg per pair of testes) at 4 °C for 1 h, followed by addition of 40 μl Protein A-Agarose beads (Roche) and incubated for an additional 3 h at 4 °C. Beads were washed three times with IP buffer (1 % Triton X-100, 150 mM NaCl, 15 mM Tris-HCl at pH 8.0, 1 mM EDTA). Immunoprecipitates were eluted with Laemmli sample buffer. Samples were fractionated on 8 % SDS–PAGE and transferred to a PVDF membrane by semi-dry transfer system (Bio-Rad). For Western analysis, membranes were probed with antibody anti-mSPO11 antibody 180 (1:2,000 in PBS containing 0.1 % Tween 20 and 5 % non-fat dry milk) overnight at 4 °C and then with horseradish peroxidase-conjugated protein A (Abcam; 1:10,000 in PBS containing 0.1 % Tween 20 and 5 % non-fat dry milk) for 2 h at room temperature. Signals were detected using the ECL+ reagent (GE Healthcare).

## Electronic supplementary material

Below is the link to the electronic supplementary material.

Supplemental Table 1 *Tg*(*Spo11*)^+/−^ mice are fertile. Adult *Tg*(*Spo11*)^+/−^ males and females (over 2 months) were tested for fertility by crossing them with a *Spo11* ^+/−^ partner. Age matching breeding between *Spo11* ^+/−^ males and females were used as control. Mice breeding were monitored over a period of 3 months from the first litter. (DOCX 55 kb)

Supplemental Table 2 *Spo11* allelic series: relative DSB levels, phenotype of chromosome synapsis and spermatogenesis elimination points (DOCX 90 kb)

Supplemental Fig. 1 Diagram of the *Spo11*-IRES-*Cre* construct was designed by inserting the Internal Ribosomal Entry (IRES)-*Cre* DNA fragment (*purple*) within Bacterial Artificial Chromosome (BAC) RP23-20N4, which contains the entire *Spo11* locus (*blue*). The IRES-*Cre* sequence was inserted downstream *Spo11* stop codon. The IRES sequence drives CRE expression. Both *Spo11* and *Cre* transcription are controlled by *Spo11* promoter. Purified *Spo11*-IRES-*Cre* BAC was microinjected into the pronuclei of fertilized eggs for conventional random insertion method. CM(R) represents the chloramphenicol resistance gene used as a probe to identify founder mice (for more details, see Pellegrini et al. 2011). (GIF 31 kb)

High resolution image (TIFF 33,272 kb)

Supplemental Fig. 2 Validation and specificity of the anti-SPO11 antibody. Immunoprecipitation and western blotting analyses of SPO11 in adult mice of the indicated genotypes. **a** The anti-SPO11 antibody (mSpo11-180) recognizes two specific bands of the expected size for SPO11β (top band; 44 kDa) and SPO11α (lower band; 40 kDa), which are not seen in the immunoprecipitation from *Spo11* ^−/−^ testis extracts. **b** The *asterisk* marks a low-mobility band likely originating from the *Spo11* knockout allele expressed in more advanced cell types, missing in *Spo11* ^−/−^ control. IgG indicates migration position of immunoglobulin heavy chains. (GIF 47 kb)

High resolution image (TIFF 7,722 kb)

Supplemental Fig. 3 DMC1 foci count in 15-dpp-old mice of the indicated genotypes [*Spo11* ^+/−^, *n* = 81 cells; *Tg*(*Spo11*)^+/−^, *n* = 50 cells; one mouse per genotype]. *p* = *p* values; one-tailed Mann-Whitney test. Lep = leptonema, Em Z = early/mid-zygonema, La Z = late zygonema. *Black bars* are means and standard deviations, *p* = *p* values (one-tailed Mann-Whitney test). (GIF 24 kb)

High resolution image (TIFF 4,856 kb)

Supplemental Fig. 4 Early/mid-zygotene-like nuclei with low foci number are morphologically similar with respect to that with high DMC1 foci count. Representative nuclear spreads of *Tg*(*Spo11*)^+/−^ spermatocytes, labeled with anti-DMC1 and anti-SYCP3 antibodies. *White arrow* points DMC1 foci. Em Z = early/mid-zygotene, Em Z-like A = early/mid-zygonema-like A cells, Em Z-like B = early/mid-zygonema-like B cells. *Scale bar* 5 μm. (GIF 356 kb)

High resolution image (TIFF 14,690 kb)
