# Supplementary material for: A surge of late-occurring meiotic double-strand breaks rescues synapsis abnormalities in spermatocytes of mice with hypomorphic expression of SPO11
Source: Chromosoma. 2015 Oct 6;125:189–203. doi: 10.1007/s00412-015-0544-7 (PMC4830894; doi:10.1007/s00412-015-0544-7)
Supplement: Supplementary file 1 — Tg(Spo11)+/− mice are fertile. Adult Tg(Spo11)+/− males and females (over 2 months) were tested for fertility by crossing them with a Spo11 +/− partner. Age matching breeding between Spo11 +/− males and females were used as control. Mice breeding were monitored over a period of 3 months from the first litter. (DOCX 55 kb) [file 412_2015_544_MOESM1_ESM.docx]

| Mating couple  Genotype | Number of  Couples | Number of  Litters | Number of  Pups | Number of  Pups/Litter |
| --- | --- | --- | --- | --- |
| *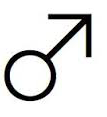* *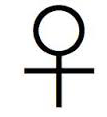*  *Spo11^-/-^ Tg(Spo11)^+/-^*  *Spo11^+/-^*  *Spo11^+/-^*   *Spo11^-/-^ Tg(Spo11)^+/-^*  *Spo11^+/-^*   *Spo11^+/-^* | 3  6  3 | 9  19  9 | 71  143  54 | 7,8  7,5  6 |

**Supplemental table 1. Faieta *et al.***
