# Supplementary material for: A surge of late-occurring meiotic double-strand breaks rescues synapsis abnormalities in spermatocytes of mice with hypomorphic expression of SPO11
Source: Chromosoma. 2015 Oct 6;125:189–203. doi: 10.1007/s00412-015-0544-7 (PMC4830894; doi:10.1007/s00412-015-0544-7)
Supplement: Supplementary file 2 — Spo11 allelic series: relative DSB levels, phenotype of chromosome synapsis and spermatogenesis elimination points (DOCX 90 kb) [file 412_2015_544_MOESM2_ESM.docx]

| Genotype | DSB levels at leptonema  vs. wt | | % of reduction | | DSB levels at early-mid  zygonema  vs. wt | | | % of reduction | | Aberrant-synapsing cell type; histological stage of arrest | References |
| --- | --- | --- | --- | --- | --- | --- | --- | --- | --- | --- | --- |
| *Spo11^+/+^*  *Spo11^+/-^*  *Spo11^-/-^Tg(Spo11βbclI)^+/+^*  *Spo11^-/-^Tg(Spo11βbclI)^+/-^*  *Spo11^-/-^Tg(Spo11)^+/-^*  *Spo11^-/-^* | | 1  0.75*  ~1^§^  0.5**  0.42  0 | | 25  0  50  58  100 | | 1  0.8*  ~1^§^  0.25**  0.6^#^  0 | 20  0  75  40  100 | | normal synapsis; no arrest  normal synapsis; no arrest  XY asynapsis, XII (complete)  pachynema-like; IV, XII (complete)  normal synapsis, no arrest^#^  zygonema-like; IV (complete) | | 1, 2  3  4  this report  5, 6, 7 |

*calculated as direct comparison to wild type (1, 2). ^§^ Compared to wild type in the same experiment; approximate value (no raw data available in ref. 3). **Inferred from raw data (DMC1 foci count) in ref. 4, using as set point, DSB levels in *Spo11^+/-^* (1, 2). ^#^It only refers to the sub-group of zygotene stage cells with no chromosome synapses defects.

**Supplemental Table 2 Faieta *et al.,***

1. Bellani, M.A., et al., *The expression profile of the major mouse SPO11 isoforms indicates that SPO11beta introduces double strand breaks and suggests that SPO11alpha has an additional role in prophase in both spermatocytes and oocytes.* Mol Cell Biol, 2010. **30**(18): p. 4391-403.

2. Cole, F., et al., *Homeostatic control of recombination is implemented progressively in mouse meiosis.* Nat Cell Biol, 2012. **14**(4): p. 424-30.

3. Kauppi, L., et al., *Distinct properties of the XY pseudoautosomal region crucial for male meiosis.* Science, 2011. **331**(6019): p. 916-20.

4. Kauppi, L., et al., *Numerical constraints and feedback control of double-strand breaks in mouse meiosis.* Genes Dev, 2013. **27**(8): p. 873-86.

5. Baudat, F., et al., *Chromosome synapsis defects and sexually dimorphic meiotic progression in mice lacking Spo11.* Mol Cell, 2000. **6**(5): p. 989-98.

6. Romanienko, P.J. and R.D. Camerini-Otero, *The mouse Spo11 gene is required for meiotic chromosome synapsis.* Mol Cell, 2000. **6**(5): p. 975-87.

7. Barchi, M., et al., *Surveillance of different recombination defects in mouse spermatocytes yields distinct responses despite elimination at an identical developmental stage.* Mol Cell Biol, 2005. **25**(16): p. 7203-15.
