# Supplementary figures and images for: A surge of late-occurring meiotic double-strand breaks rescues synapsis abnormalities in spermatocytes of mice with hypomorphic expression of SPO11
Source: Chromosoma. 2015 Oct 6;125:189–203. doi: 10.1007/s00412-015-0544-7 (PMC4830894; doi:10.1007/s00412-015-0544-7)

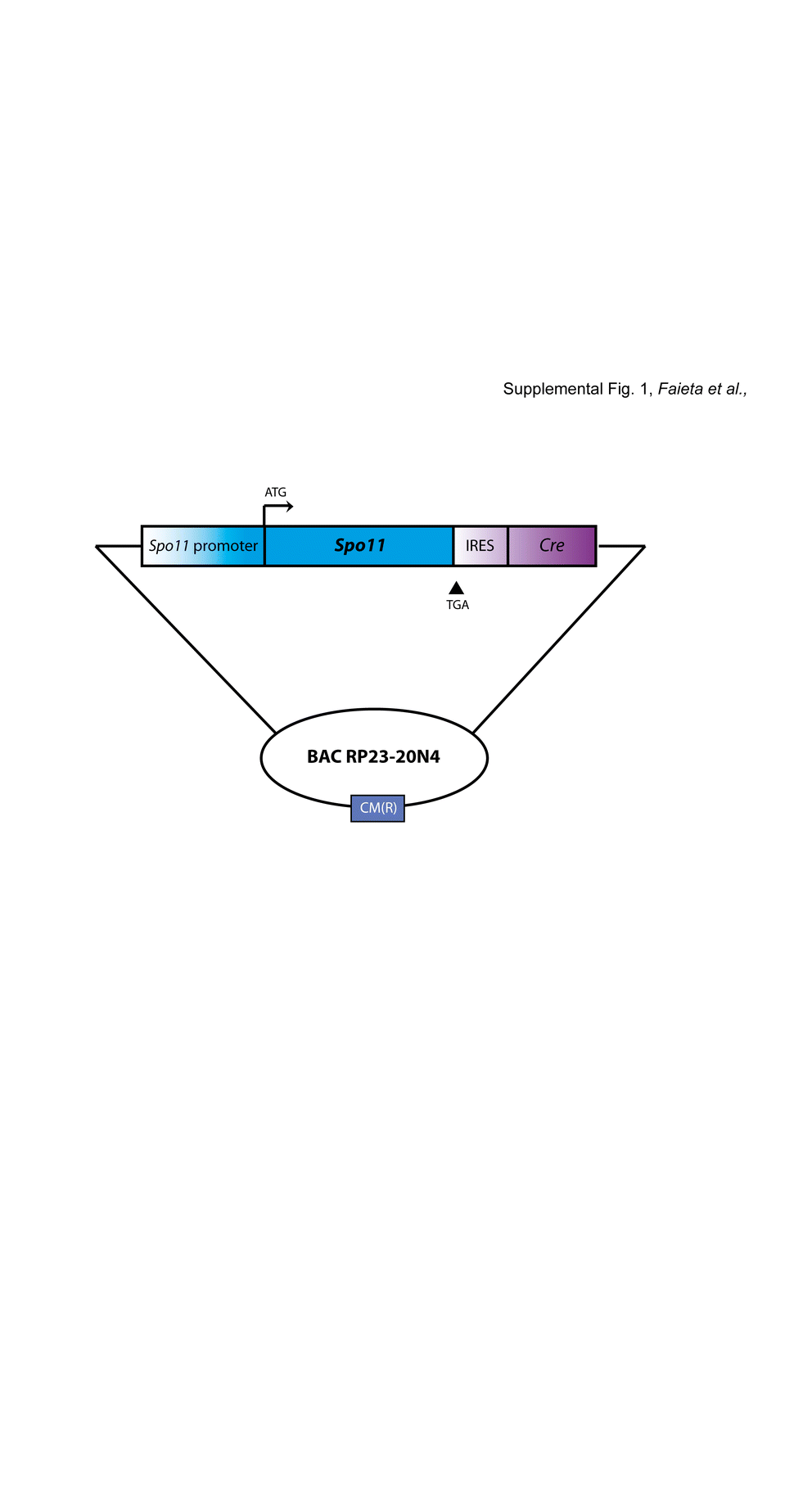

Supplement: Supplementary file 3 — Diagram of the Spo11-IRES-Cre construct was designed by inserting the Internal Ribosomal Entry (IRES)-Cre DNA fragment (purple) within Bacterial Artificial Chromosome (BAC) RP23-20N4, which contains the entire Spo11 locus (blue). The IRES-Cre sequence was inserted downstream Spo11 stop codon. The IRES sequence drives CRE expression. Both Spo11 and Cre transcription are controlled by Spo11 promoter. Purified Spo11-IRES-Cre BAC was microinjected into the pronuclei of fertilized eggs for conventional random insertion method. CM(R) represents the chloramphenicol resistance gene used as a probe to identify founder mice (for more details, see Pellegrini et al. 2011). (GIF 31 kb) [file 412_2015_544_Fig8_ESM.gif]

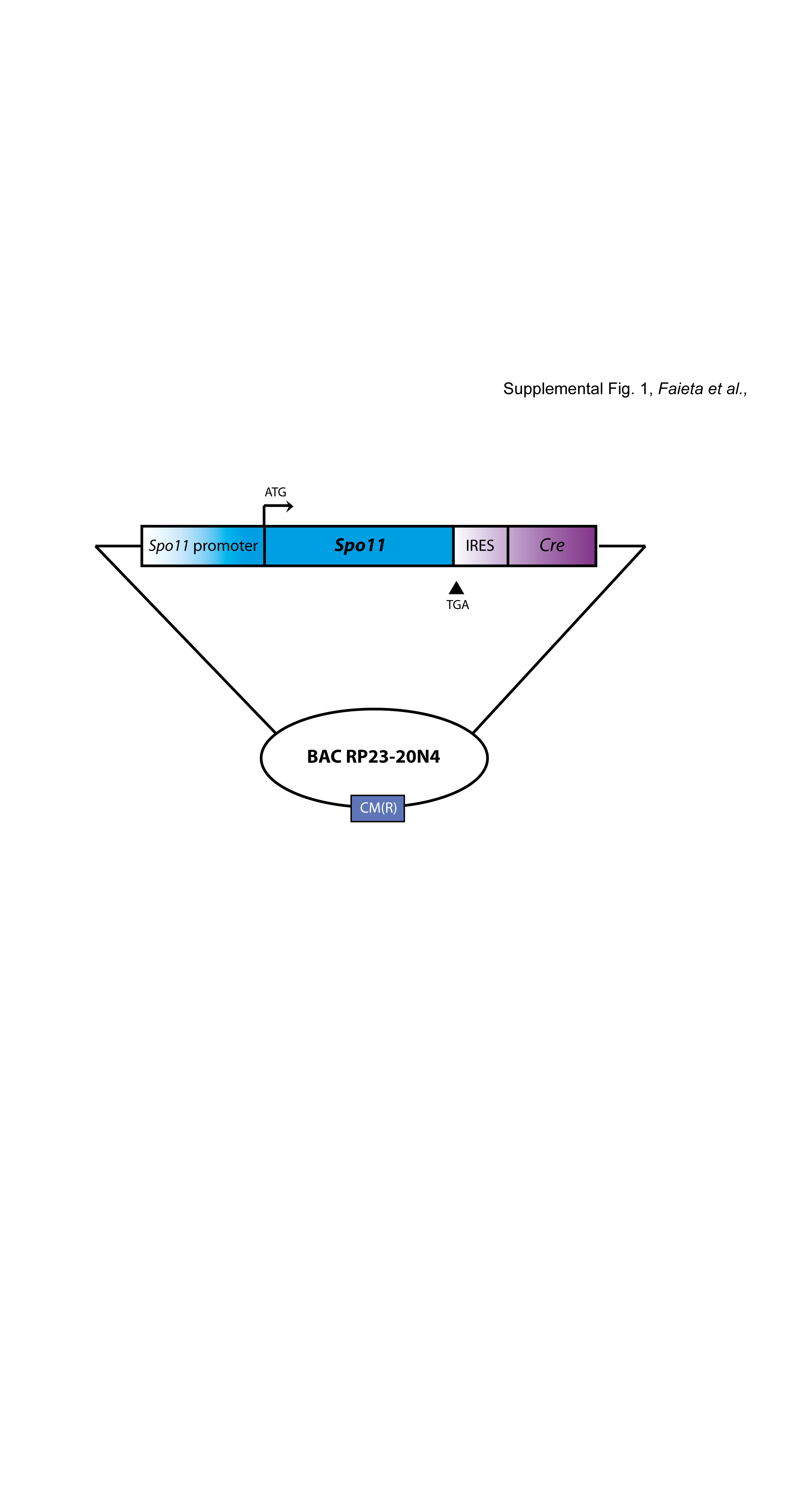

Supplement: Supplementary file 4 — High resolution image (TIFF 33,272 kb) [file 412_2015_544_MOESM3_ESM.tif]

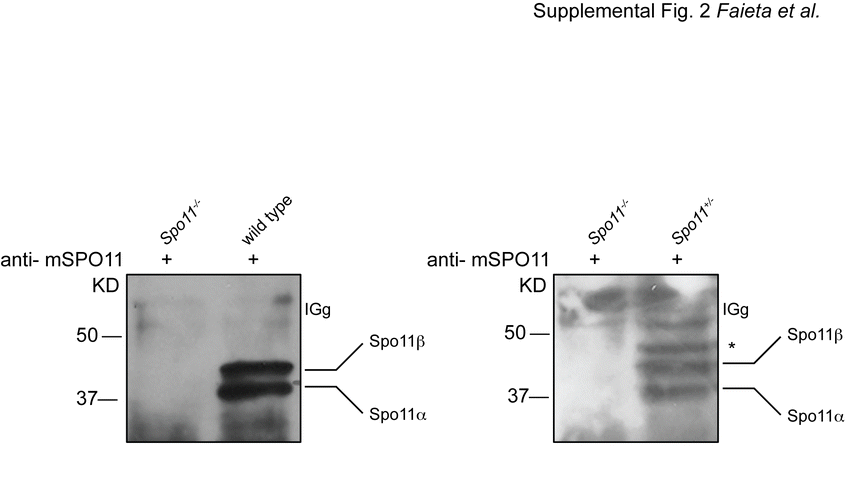

Supplement: Supplementary file 5 — Validation and specificity of the anti-SPO11 antibody. Immunoprecipitation and western blotting analyses of SPO11 in adult mice of the indicated genotypes. a The anti-SPO11 antibody (mSpo11-180) recognizes two specific bands of the expected size for SPO11β (top band; 44 kDa) and SPO11α (lower band; 40 kDa), which are not seen in the immunoprecipitation from Spo11 −/− testis extracts. b The asterisk marks a low-mobility band likely originating from the Spo11 knockout allele expressed in more advanced cell types, missing in Spo11 −/− control. IgG indicates migration position of immunoglobulin heavy chains. (GIF 47 kb) [file 412_2015_544_Fig9_ESM.gif]

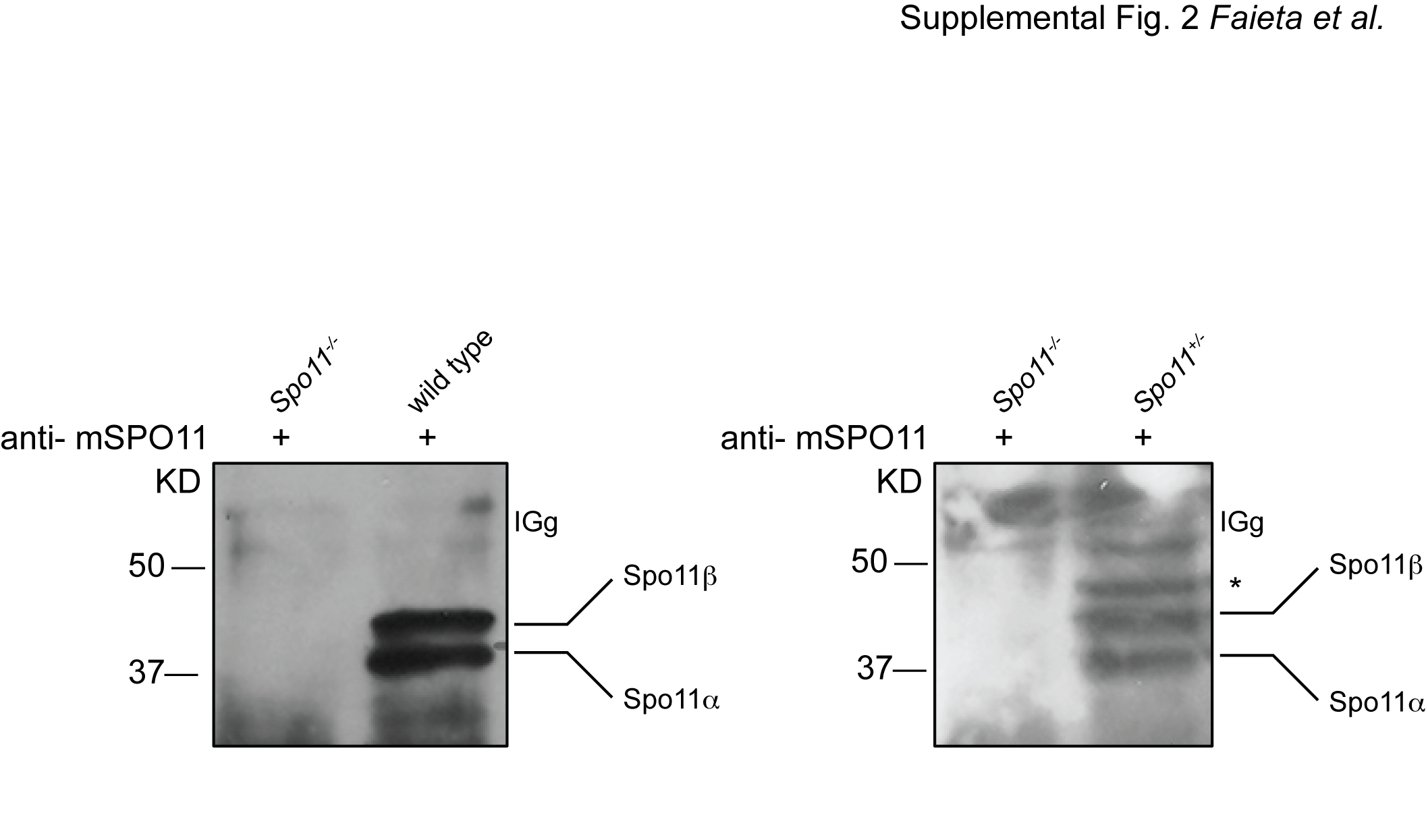

Supplement: Supplementary file 6 — High resolution image (TIFF 7,722 kb) [file 412_2015_544_MOESM4_ESM.tif]

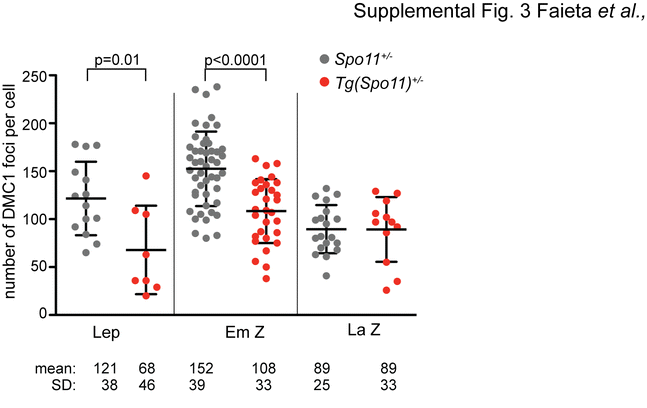

Supplement: Supplementary file 7 — DMC1 foci count in 15-dpp-old mice of the indicated genotypes [Spo11 +/−, n = 81 cells; Tg(Spo11)+/−, n = 50 cells; one mouse per genotype]. p = p values; one-tailed Mann-Whitney test. Lep = leptonema, Em Z = early/mid-zygonema, La Z = late zygonema. Black bars are means and standard deviations, p = p values (one-tailed Mann-Whitney test). (GIF 24 kb) [file 412_2015_544_Fig10_ESM.gif]

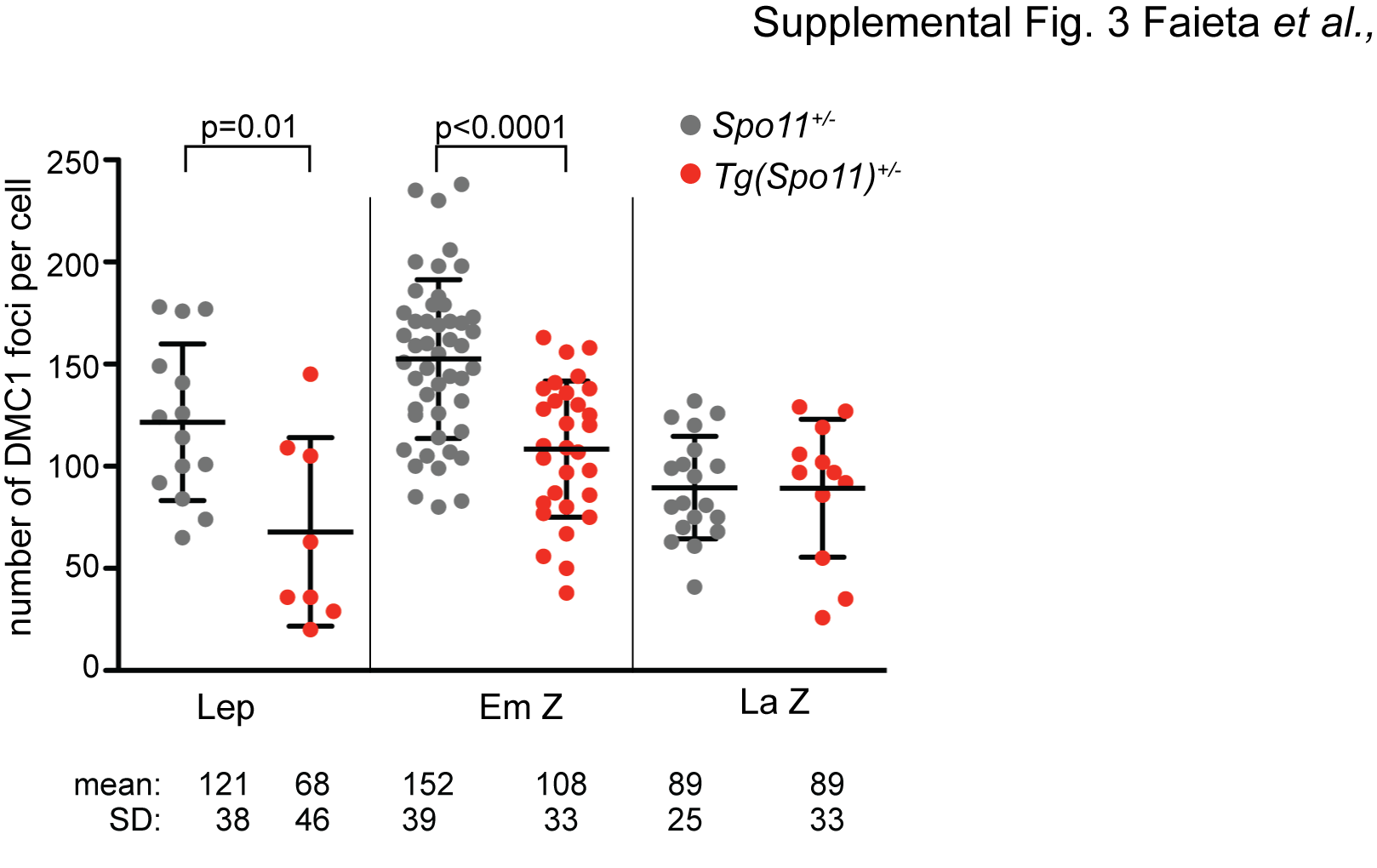

Supplement: Supplementary file 8 — High resolution image (TIFF 4,856 kb) [file 412_2015_544_MOESM5_ESM.tif]

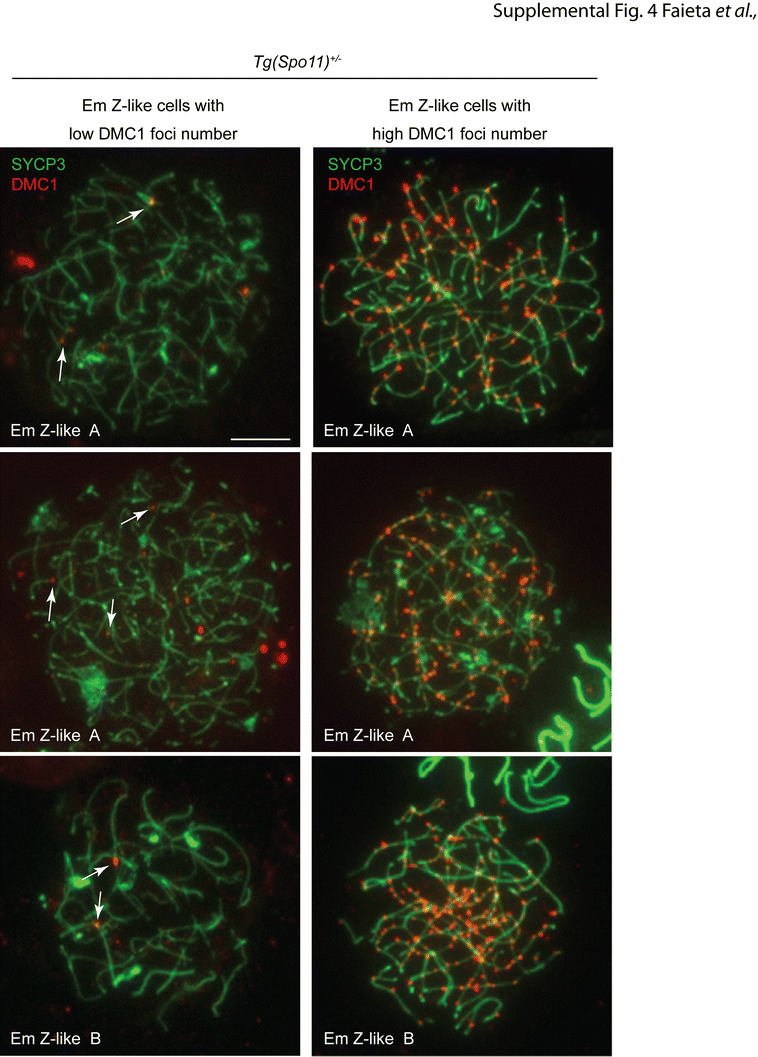

Supplement: Supplementary file 9 — Early/mid-zygotene-like nuclei with low foci number are morphologically similar with respect to that with high DMC1 foci count. Representative nuclear spreads of Tg(Spo11)+/− spermatocytes, labeled with anti-DMC1 and anti-SYCP3 antibodies. White arrow points DMC1 foci. Em Z = early/mid-zygotene, Em Z-like A = early/mid-zygonema-like A cells, Em Z-like B = early/mid-zygonema-like B cells. Scale bar 5 μm. (GIF 356 kb) [file 412_2015_544_Fig11_ESM.gif]

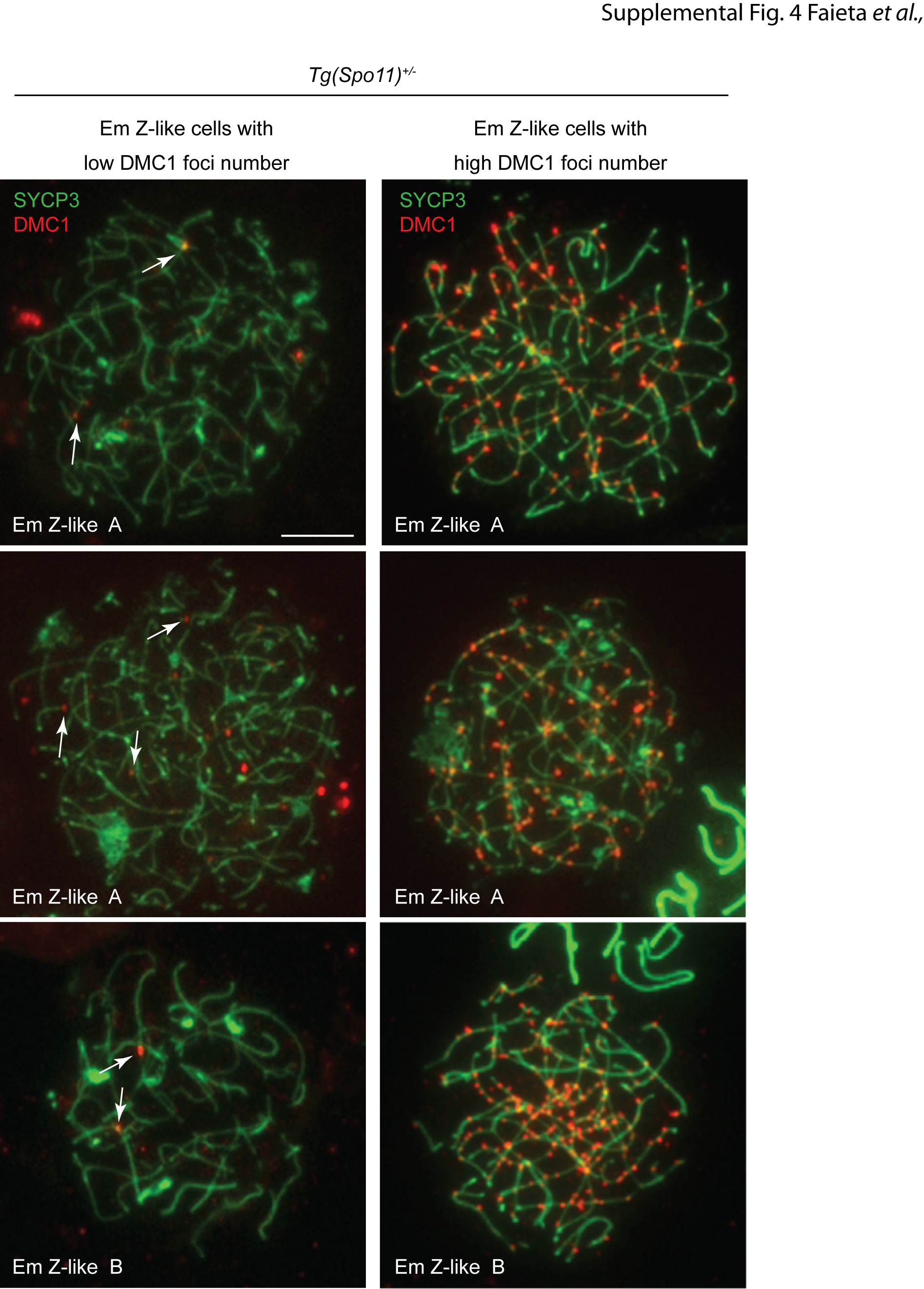

Supplement: Supplementary file 10 — High resolution image (TIFF 14,690 kb) [file 412_2015_544_MOESM6_ESM.tif]
